# Microbial solutions for a changing world

**DOI:** 10.1093/sumbio/qvag011

**Published:** 2026-07-07

**Authors:** Zahra F Islam, Paul Sainsbury, Chris Greening

**Affiliations:** School of Agriculture, Food and Ecosystem Sciences, The Faculty of Science, The University of Melbourne, Parkville, Victoria 3010, Australia; ARC Research Hub for Smart Fertilisers, The University of Melbourne, Parkville, Victoria 3010, Australia; Applied Microbiology International, Cambridge CB1 2LA, United Kingdom; Department of Microbiology, Biomedicine Discovery Institute, Monash University, Clayton, Victoria 3800, Australia; Securing Antarctica’s Environmental Future, Monash University, Clayton, Victoria 3800, Australia

**Keywords:** biogeochemical cycles, climate, bioremediation, One Health, ecosystem functioning

## Abstract

*Microbial Solutions for a Changing World* was an inaugural international conference hosted at Monash University, Melbourne, and presented by Applied Microbiology International. The meeting brought together researchers from across the Asia-Pacific region to examine how microbiological research informs responses to climate-related challenges across environmental, health, and applied contexts. Recognizing the central role of microorganisms in regulating greenhouse gas fluxes, shaping disease dynamics, and underpinning ecosystem function, it provided a forum for focused exchange at the intersection of microbiology and climate research. The programme was organized around three thematic areas: greenhouse gas cycling and mitigation, health impacts and pathogen control, and environmental impacts and ecosystem restoration. Plenary lectures and contributed presentations addressed microbial processes operating across managed, natural, and extreme environments, alongside discussion of how microbiological insight can inform mitigation, disease management, and restoration strategies under changing environmental conditions. Early-career researchers were placed at the centre of the programme, with structured activities aimed at supporting interaction and collaboration. By convening researchers working across diverse sub-disciplines, the conference highlighted common challenges and opportunities in aligning microbiology with climate response efforts. *Microbial Solutions for a Changing World* demonstrated the value of dedicated forums for advancing sustainable microbiology within the Asia-Pacific region and beyond.

**Sustainability Statement**

This meeting report primarily contributes to Sustainable Development Goal 13 (Climate Action), with particular relevance to Targets 13.1 and 13.3, by examining how microbiological research can strengthen understanding of climate-related risks and inform practical responses. The conference addressed microbial processes relevant to greenhouse gas cycling, climate-sensitive disease dynamics, and ecosystem recovery, alongside discussion of emerging microbiology-based interventions and routes towards application. Through focused exchange across environmental, health, and applied microbiology, the meeting supported knowledge sharing and capacity building relevant to climate change mitigation and adaptation. Additional intersections with SDG 3 (Good Health and Well-Being), SDG 12 (Responsible Consumption and Production), SDG 14 (Life Below Water), and SDG 15 (Life on Land) were also identified.

## Introduction

Human activity is altering climate, land use, and aquatic systems in ways that reshape microbial communities and the processes they mediate. These changes matter because microorganisms regulate greenhouse gas fluxes, influence host–pathogen interactions, and underpin ecosystem function in soils and waters (Cavicchioli et al. 2019). Shifts in microbial biodiversity and activity therefore have consequences that extend beyond microbiology, affecting resilience in managed and natural systems, and shaping the feasibility of climate response measures.

The Asia-Pacific region includes ecosystems that are highly exposed to climate-related pressures, alongside rapidly changing agricultural and urban environments. In this context, there is a practical need for forums that bring together microbiologists working across environmental, health, and applied domains to address shared scientific questions and to identify points of connection between mechanism, intervention, and implementation (Tiedje et al. 2022, Peixoto et al. 2024). Despite increasing interest in “sustainability” as a framing concept, dedicated opportunities for focused exchange at the intersection of microbiology and climate research have remained limited, particularly within regional networks (Oliveira et al. 2023).

*Microbial Solutions for a Changing World* was established to address this gap. The conference was developed under the academic leadership of Professor Chris Greening (Monash University) as an initiative of Applied Microbiology International and hosted at Monash University, Melbourne. Chaired by Dr Zahra Islam (University of Melbourne) and supported by the Australian Society for Microbiology, the meeting aimed to convene researchers across relevant sub-disciplines to examine shared challenges and to identify areas for collaboration. The programme positioned early-career researchers (ECRs) centrally, with the explicit intention of increasing visibility for emerging ECRs and strengthening professional connections within the community (Krinos et al. 2025).

## Conference scope and thematic structure

Addressing the role of microbiology in climate change requires engagement across multiple spatial scales and application domains, from molecular and physiological processes to ecosystem-level outcomes and human health (Cavicchioli et al. 2019). In recognition of this breadth, the conference was structured to bring together research communities that do not always interact directly, but whose work is increasingly shaped by shared environmental pressures.

The programme was organized around three thematic areas: greenhouse gas cycling and mitigation, health impacts and pathogen control, and environmental impacts and ecosystem restoration. These themes were selected to reflect areas in which climate change is already influencing microbial processes and where microbiological research is contributing to emerging responses. Together, they provided a framework for examining how fundamental understanding of microbial function can inform applied strategies under changing environmental conditions.

Each theme combined plenary lectures with contributed presentations and extended discussion, allowing participants to situate specific research findings within broader conceptual and applied contexts. Rather than treating these areas as discrete, discussions frequently highlighted points of overlap, particularly where microbial processes link biogeochemical change with host health and ecosystem resilience. This structure encouraged comparison across systems and supported dialogue between researchers working in environmental, clinical, agricultural, and applied microbiology. Informal networking sessions alongside the Hackathon-like ECR focused workshop served as the major platform for discussions between participants between scientific sessions.

The thematic organization also provided a basis for identifying common challenges that cut across sub-disciplines, including the need to link microbial-scale processes with system-level outcomes, to integrate laboratory and field-based evidence, and to consider how microbiological knowledge can inform interventions that are both effective and environmentally responsible (Fady 2025). These cross-cutting issues recurred throughout the meeting and informed subsequent discussions on future research directions and collaboration.

## Greenhouse gas cycling and mitigation

Microbial processes regulate the production and consumption of key greenhouse gases, particularly methane and nitrous oxide, making microbiology central to both climate prediction and mitigation (Stein 2020). Sessions within this theme examined how microbial activity responds to environmental change across managed, natural, and extreme environments, and how this knowledge can inform mitigation strategies.

In a plenary lecture, Professor Liu Ye (*Flushing Out Emissions*) examined microbial controls on greenhouse gas fluxes in managed environments, emphasizing the need to link microbial-scale processes with direct emissions measurements. Her presentation highlighted how integrating microbiological data with system-level monitoring can improve the evaluation of mitigation interventions (Li et al. 2025). Dr Sinéad Leahy (*Microbes, Methane and the Future of Farming*) focused on agricultural systems, discussing microbiological approaches to reducing methane emissions while maintaining productivity. Her contribution underscored the importance of understanding microbial function within complex host–environment interactions (Leahy et al. 2022). In a further plenary, Professor Sung-Keun Rhee (*Microbial Crossroads of Methane and Nitrous Oxide in Extreme Environments*) explored microbial pathways governing greenhouse gas cycling under extreme physicochemical conditions, drawing attention to environments that are often underrepresented in climate models (Awala et al. 2024).

Contributed presentations extended these perspectives through experimental studies, field-based measurements, and genomic analyses examining microbial greenhouse gas dynamics. Highlights included talks spanning fundamental analysis of both enzymatic mediators and mitigators of greenhouse gases in high-emitting systems, investigations into understudied ecosystems like tree bark and Blue Carbon wetlands as new frontiers for greenhouse gas consumption and novel biotechnological advances to utilize waste gases within a circular economy (Rodriguez et al. 2025, Leung et al. 2026). Across sessions, discussions highlighted the challenge of translating mechanistic insight into mitigation strategies that remain effective across heterogeneous landscapes, and the importance of integrating microbiology with land management and policy frameworks.

## Health impacts and pathogen control

Climate-driven environmental change is reshaping the distribution and behaviour of microbial pathogens, with implications for human, animal, and wildlife health. Sessions within this theme addressed how altered ecological conditions influence host–pathogen interactions and how microbiological research can inform responses to emerging disease risks (Zinsstag et al. 2018).

In a plenary presentation, Dr Fran Short (*Combatting Infectious Disease in a Changing World*) examined how environmental stressors such as biocides affect pathogen dynamics and host susceptibility, considering consequences for disease emergence under changing climatic conditions (Cartledge et al. 2025). To highlight the need to move towards a One Health model, plenary Dr Rebecca Webb (*Microbial Tools for Wildlife Disease Resilience*) discussed microbiological approaches to supporting disease resilience in wildlife populations exposed to environmental disturbance, highlighting the role of microbiology in conservation-oriented disease management (Berger et al. 2024). The final plenary, Associate Professor Bart Eijkelkamp (*Impact of Diet on Bacterial Infections*), explored links between host nutrition, bacterial physiology, and infection outcomes, illustrating how environmental and dietary factors shape microbial behaviour within hosts (Waters and Eijkelkamp 2024).

Broader discussions considered how microbiological research can be integrated into disease control strategies under changing environmental conditions. Presentations addressed the application of One Health frameworks and developments in diagnostic and surveillance approaches, including the need to explore a wide range of ecosystems for novel bioactive compounds to address the burgeoning antimicrobial resistance crisis (Negeri et al. 2025). The talks further emphasized the need to connect fundamental investigative microbiology approaches with ecological and public health perspectives when responding to climate-associated disease threats.

## Environmental impacts and ecosystem restoration

Microorganisms underpin ecosystem function and play a critical role in recovery following environmental disturbance. This theme focused on how microbiology can inform restoration strategies and sustainable resource use across terrestrial and aquatic systems.

In a plenary lecture, Dr Manpreet Dhami (*I ndigenous Partnerships and Microbial Science for Ecological Restoration*) highlighted the value of integrating microbiological research with Indigenous knowledge systems and long-term land management practices (Rowe et al. 2025). Her presentation emphasized the importance of place-based approaches that recognize microbial processes as part of broader ecological relationships. Dr Ezequiel Santillan (*Engineering Microbial Futures for a Circular Bioeconomy*) examined the potential of engineered microbial systems to support circular bioeconomy applications, considering both technical opportunities and sustainability constraints (Thi et al. 2026). In the final conference plenary, distinguished Professor Brajesh Singh (*Soil Biodiversity for a Sustainable Planet*) discussed the role of soil microbial diversity in maintaining ecosystem function under environmental pressure, drawing attention to links between biodiversity loss and reduced resilience (Singh et al. 2025).

Contributed sessions addressed microbiological applications in bioremediation and ecosystem recovery across degraded terrestrial landscapes and impacted aquatic environments. Discussions emphasized that effective restoration depends on maintaining functional microbial communities and tailoring interventions to local ecological conditions, particularly where long-term resilience or the generation of effective probiotic interventions are the central objectives (Obregon et al. 2025, Matthews et al. 2026).

## Conference legacy

Beyond its immediate outputs, the inaugural *Microbial Solutions for a Changing World* meeting demonstrated how scientific conferences can function as sites of disciplinary development rather than serving only as venues for the presentation of completed work. By structuring discussion around greenhouse gas cycling, infectious disease, and ecosystem restoration, the meeting positioned sustainability as a central concern shaping contemporary microbiology, influencing how research questions are framed and how microbiological knowledge is situated in relation to societal challenges (Crowther et al. 2024). Additionally, an interactive Hackathon-like workshop centred around translating microbiological research into policy and real-world solutions was included to pair renowned experts in this space with ECRs to equip them with the knowledge to translate their fundamental research into practice.

The meeting was also notable for its emphasis on early-career leadership and inclusive participation. By placing early-career researchers at the centre of the programme while maintaining high scientific standards, the conference challenged assumptions about how excellence is structured within scientific meetings. This approach supported substantive exchange across sub-disciplines and strengthened regional networks within the Asia-Pacific microbiology community, while remaining outward-looking in scope.

Taken together, the discussions generated momentum for continued collaboration beyond the event itself. Participants identified shared challenges that extend across the three thematic areas, including the need for stronger links between microbiological research and decision-making processes, improved support for capacity building in underrepresented regions, and clearer pathways for translating microbiological insight into practice in ways consistent with sustainability objectives (Lee and Kirby 2019, Crowther et al. 2024, Williams and Lynch 2025). These priorities have informed early planning for future activities intended to support sustained engagement rather than one-off outputs.

The success of the inaugural meeting provides a foundation for the development of an ongoing conference series with a commitment to global inclusion. Future events are anticipated in regions where access to international scientific platforms remains uneven, particularly across parts of Asia, Africa, and the Pacific. By positioning the conference as part of the wider infrastructure of the field, *Microbial Solutions for a Changing World* offers a model for how applied microbiology can align scientific rigour with responsibility over the longer term.

## Data Availability

No new data were generated or analysed in this article.
